# Statement on imaging and pulmonary hypertension from the Pulmonary Vascular Research Institute (PVRI)

**DOI:** 10.1177/2045894019841990

**Published:** 2019-09-06

**Authors:** David G. Kiely, David L. Levin, Paul M. Hassoun, Dunbar Ivy, Pei-Ni Jone, Jumaa Bwika, Steven M. Kawut, Jim Lordan, Angela Lungu, Jeremy A. Mazurek, Shahin Moledina, Horst Olschewski, Andrew J. Peacock, G.D. Puri, Farbod N. Rahaghi, Michal Schafer, Mark Schiebler, Nicholas Screaton, Merryn Tawhai, Edwin J.R. van Beek, Anton Vonk-Noordegraaf, Rebecca Vandepool, Stephen J. Wort, Lan Zhao, Jim M. Wild, Jens Vogel-Claussen, Andrew J. Swift

**Affiliations:** 1 Sheffield Pulmonary Vascular Disease Unit, Royal Hallamshire Hospital, Sheffield, UK; 2 Department of Infection, Immunity and Cardiovascular Disease and Insigneo Institute, University of Sheffield, Sheffield, UK; 3 Department of Radiology, Mayo Clinic, Rochester, MN, USA; 4 Department of Medicine John Hopkins University, Baltimore, MD, USA; 5 Paediatric Cardiology, Children’s Hospital, University of Colorado School of Medicine, Denver, CO, USA; 6 Aga Khan University, Nairobi, Kenya; 7 Department of Medicine, Perelman School of Medicine at the University of Pennsylvania, Philadelphia, PA, USA; 8 Freeman Hospital, Newcastle Upon Tyne, Newcastle, UK; 9 Technical University of Cluj-Napoca, Cluj-Napoca, Romania; 10 Division of Cardiovascular Medicine, Hospital of the University of Pennsylvania, Philadelphia, PA, USA; 11 Great Ormond Street Hospital, London, UK; 12 Division of Pulmonology, Ludwig Boltzmann Institute Lung Vascular Research, Graz, Austria; 13 Scottish Pulmonary Vascular Disease, Unit, University of Glasgow, Glasgow, UK; 14 Department of Anaesthesiology and Intensive Care, Post Graduate Institute of Medical Education and Research, Chandigarh, India; 15 Brigham and Women’s Hospital, Harvard Medical School, Boston, MA, USA; 16 Department of Radiology, University of Wisconsin School of Medicine and Public Health, Madison, WI, USA; 17 Royal Papworth Hospital NHS Trust, Papworth Everard, UK; 18 Auckland Bioengineering Institute, Auckland, New Zealand; 19 Edinburgh Imaging, Queens Medical Research Institute, University of Edinburgh, Edinburgh, UK; 20 University Medical Centre, Amsterdam, the Netherlands; 21 University of Arizona, Division of Translational and Regenerative Medicine, Tucson, AZ, USA; 22 Royal Brompton Hospital, London, UK; 23 Imperial College, London, UK; 24 Academic Department of Radiology, University of Sheffield, Sheffield, UK; 25 Institute of diagnostic and Interventional Radiology, Medical Hospital Hannover, Hannover, Germany

**Keywords:** pulmonary hypertension, diagnosis, imaging, computed tomography, magnetic resonance imaging, echocardiography, guidelines, cardiac catheterization, algorithm

## Abstract

Pulmonary hypertension (PH) is highly heterogeneous and despite treatment advances it remains a life-shortening condition. There have been significant advances in imaging technologies, but despite evidence of their potential clinical utility, practice remains variable, dependent in part on imaging availability and expertise. This statement summarizes current and emerging imaging modalities and their potential role in the diagnosis and assessment of suspected PH. It also includes a review of commonly encountered clinical and radiological scenarios, and imaging and modeling-based biomarkers. An expert panel was formed including clinicians, radiologists, imaging scientists, and computational modelers. Section editors generated a series of summary statements based on a review of the literature and professional experience and, following consensus review, a diagnostic algorithm and 55 statements were agreed. The diagnostic algorithm and summary statements emphasize the key role and added value of imaging in the diagnosis and assessment of PH and highlight areas requiring further research.

## Introduction

Pulmonary hypertension (PH) is highly heterogeneous, is challenging to diagnose and treat, and has a survival worse than many forms of common cancer.^1,2^ It ranges from a rare form, pulmonary arterial hypertension (PAH), characterized by a vasculopathy and frequently severe elevation of pressure, to more common usually mild elevations of pulmonary artery pressure (PAP) seen in severe cardiac and respiratory disease.^3,4^ The current system of classification identifies five groups, each with distinct pathophysiological characteristics.^2,3,5^ The diagnosis of PH is usually first suggested by echocardiography or chest radiography with confirmation of an elevated PAP at right heart catheterization (RHC). Phenotyping is based on a careful history, blood testing for associated conditions, detailed physiological, and imaging investigations.

The treatment of PH is dependent on the underlying cause. For patients with PAH, drug therapy targeting imbalances in vasoconstrictor and vasodilator mediators have been shown to improve exercise capacity, quality of life, and event-free survival.^6–13^ However, PAH remains a life-limiting and debilitating condition. In chronic thromboembolic pulmonary hypertension (CTEPH), pulmonary endarterectomy is an established technique with excellent long-term outcomes^14–18^ and, more recently, drug therapy^19–21^ and balloon pulmonary angioplasty (BPA)^22,23^ have shown benefit in selected groups of patients with CTEPH. For patients with other forms of PH, such as in association with cardiac and respiratory disease, trials of PAH therapies have thus far been disappointing.^24–26^ Accurate classification is key, not only as it defines treatment but also prognosis^27–29^ and careful assessment is therefore crucial in the assessment of patients with suspected PH.

Current assessment tools in the PH clinic and endpoints used in clinical trials may be limited by a number of factors. These include insensitivity to change, lack of repeatability, and the invasive nature of tests. There is a need to identify new tools and endpoints to aid the physician both in the clinical environment and in studies of new interventions.^30–36^ Importantly, over the last 20 years, there has been major advances in imaging techniques and their application including the use of echocardiography, nuclear medicine, computed tomography (CT) scanning, magnetic resonance imaging (MRI), and molecular imaging. There is growing evidence demonstrating the value of various imaging modalities in the classification, risk stratification, and follow-up of patients with PH. Imaging studies have also provided insights into pathophysiological mechanisms.^37–45^

The use of imaging varies across the globe due to a variety of factors including personal preference, availability, and cost. Given the complex nature of certain imaging investigations, there are also differences in methods of scan acquisition and post-processing within a given modality. Consequently, the Pulmonary Vascular Research Institute (PVRI) have identified imaging as an important area for international collaboration, with the aim of developing evidence-based statements and sharing best practice, while recognizing that approaches need to be tailored to imaging availability. This statement on imaging in PH is aimed at physicians (including cardiologists and pulmonologists), PH specialists, radiologists, and imaging scientists.

## Methods

The PVRI Imaging Task force met for the first time in Rome in 2016 with an aim of improving imaging practice globally in PH. A summary statement from the PVRI was identified as an important first step in achieving this goal. Participants were invited from the existing PVRI membership in addition to international imaging experts. The group included representatives from wide-ranging professional backgrounds and different geographical areas with varied access to imaging.

Groups of authors were assigned to specific sections to review current literature, identify summary statements, and develop a diagnostic algorithm. An editorial board met to preview and refine summary statements and ensure uniformity of style (DGK, DL, JVC, and AJS). To be included in the final document, summary statements required agreement of 80% of authors. Statements not meeting this requirement were reworded until this threshold was reached or the statement rejected. Given the rapid development of imaging technologies, the recommendations reflect a combination of published evidence, current practice, and expert opinion.

All authors read and approved the manuscript before submission.

## Section 1: Imaging modalities used in the assessment of PH

### 1.1 Chest radiography

#### Summary statements

1. A chest radiograph is recommended as the initial imaging test in the assessment of unexplained breathlessness.
2. A normal chest radiograph does not exclude the diagnosis of PH
3. Features of PH include pulmonary artery (PA) enlargement and cardiomegaly.

#### Chest radiography

Patients with PH frequently present with breathlessness. The principal role of the chest radiograph (CXR) is to identify other common causes of breathlessness (e.g. parenchymal lung disease, pneumonia, pulmonary edema, pleural effusion, and pneumothorax). The findings of PH on CXR vary. There may be enlargement of the central pulmonary arteries with pruning of the peripheral vessels, features that were observed in the majority of patients in a registry of patients with idiopathic PAH.^46^ In addition, cardiomegaly and features suggestive of right atrial enlargement may be observed. However, a normal CXR cannot exclude the diagnosis and the CXR may be normal, where PAP elevation is modest. Radiographic features may also suggest the cause of PH, such as upper lobe venous diversion and left atrial enlargement in patients with left heart disease, vascular plethora, and peripheral pruning in patients with Eisenmenger physiology, and interstitial opacities in patients with diffuse parenchymal lung disease.

### 1.2: Echocardiography

#### Summary statements

1. Echocardiography is the test of choice in the initial evaluation of suspected PH.
2. Echocardiography should include an assessment of PAP, cardiac, and valvular function.
3. A probability of PH should be generated using estimated PAP and additional echocardiographic features.

Doppler and two-dimensional (2D) echocardiography remains the screening test of choice in the evaluation of suspected PH.^47–50^ While right ventricular systolic pressure (RVSP) from the continuous wave Doppler of the peak tricuspid regurgitant velocity (TRV) is the most well-known tool for assessing the presence or absence of PH, this metric can be subject to several limitations resulting in over or under estimation of the true pulmonary artery systolic pressure (PASP).^51^ Over the last decade, there have been many studies evaluating the role of clinically useful and readily available echo-Doppler parameters that allow one to move beyond the PASP and assess not just the likelihood of PH, but also the hemodynamic underpinnings of the disease (i.e. left heart disease vs. PAH).^52,53^ As noted in the most recent European Society of Cardiology (ESC)/European Respiratory Society (ERS) PH guidelines, “echocardiography should always be performed when pulmonary hypertension is suspected and may be used to infer a diagnosis of pulmonary hypertension in patients in whom multiple different echocardiographic measurements are consistent with this diagnosis,” even in the absence of an elevated TRV.^54^ This can then be used to generate a probability of PH which will inform the diagnostic strategy and “decide the need for cardiac catheterization in individual patients.”^54^ Furthermore, several echo-Doppler parameters have been shown to have prognostic value in the setting of established PH.

Clinically useful measures of PH (i.e. elevated RV afterload) on echocardiography include characteristics of the RV outflow tract (RVOT) pulse wave Doppler envelope such as a reduced acceleration time (<100 ms),^53,55–57^ systolic notching,^53,58,59^ and pulmonary insufficiency velocity to estimate mean PAPs (mPAP), as well as interventricular septal flattening (as characterized by the eccentricity index),^60^ increased right-to-left ventricular ratio (0.8–1.0, 1.1–1.4, and ≥1.5 corresponding to mild, moderate, and severe RV dilatation, respectively),^52,54^ RV hypertrophy and right atrial dilation^61^ and measures of RV function including tricuspid annular plane systolic excursion (TAPSE),^62,63^ and RV fractional area change (RVFAC).^63–65^ While RVFAC is often limited in the setting of severe RV enlargement,^52,63,66^ TAPSE is a reproducible measure of RV function which measures the total displacement of the heart from the RV base toward the apex in systole, and correlates with radionuclide-derived RV ejection fraction (RVEF).^63^ TAPSE has also been shown to be prognostic of poor outcome in PH in all-comers,^62^ as well as in follow-up assessment in a PAH population after initiation of PH therapy.^67,68^ However, TAPSE has not been shown as an effective marker of RV function in pediatric PH.^69^ Other echocardiographic measures include the myocardial performance (Tei) index^70^ and S’ obtained from tissue Doppler imaging of the tricuspid annulus.^71^ Markers of adverse outcomes include pericardial effusion and enlarged right atrium.^72^ More recently, RV longitudinal strain using 2D speckle tracking has been employed in the quantification of RV function in patients with PH and has been shown to be impaired in patients with PH, a predictor of mortality^73–76^ and useful for assessment of therapy response.^77,78^ Lastly, given the complex anatomy of the right ventricle, recent investigation has focused on the use of three-dimensional (3D) echocardiography^79^ and strain to assess global and regional RV structure and function and predict outcomes in PH.^80^

### 1.3 Nuclear medicine imaging

#### Summary statements

1. A normal perfusion single photon emission computed tomography (SPECT) excludes chronic thromboembolic disease that will benefit from pulmonary endarterectomy and BPA.
2. Ventilation and perfusion SPECT or SPECT CT is superior to planar scintigraphy.
3. In unexplained hypoxemia, a nuclear medicine shunt assessment can be used to identify the presence of a right-to-left shunt.
4. In patients with suspected PA sarcoma, positron-emission tomography (PET) is recommended.

Ventilation/perfusion (V/Q) SPECT is recommended by the ESC as the first line screening test for patients with CTEPH.^1^ The technique is well established and has excellent diagnostic value particularly in the absence of lung disease. The perfusion image involves exposure to ionizing radiation and requires injection of 99mTc labelled macroaggregated human albumin (10–90 µm in diameter).^20^ The macro-aggregated albumin becomes trapped within the small pulmonary arterioles and a 3D image of pulmonary perfusion is acquired. In CTEPH, typically peripheral wedge-shaped defects of varying size are shown. Mismatch to ventilation can be confirmed by comparing ventilation and perfusion images. The added value of performing ventilation imaging is debated and in many centers perfusion imaging alone is performed and compared to CT which better demonstrates parenchymal lung disease. CTEPH may be missed on CT as attenuated distal vessels, subsegmental stenosis, and webs may not be appreciated. Early studies demonstrated that scintigraphy was more sensitive than CT for the detection of CTEPH. However, given advances in technology, more recent studies have shown that CT pulmonary angiography and CT SPECT techniques are equally sensitive.^21–23^ Dual-energy CT (DECT) or CT with iodine mapping allow construction of relative perfusion maps in addition to providing angiographic images.^81–87^ Given that 99mTc labelled macroaggregates are trapped by small pulmonary arterioles, the presence of radioactive uptake in organs supplied by the systemic circulation (e.g. the kidneys) can be used to identify the presence of a right-to-left shunt in hypoxemic patients with PH.^19^

Positron-emission tomography (PET) allows for observation of metabolic activity in the body which can be reconstructed to produce 3D images. Fluorodeoxyglucose (FDG), a glucose analogue, can be used to assess glucose uptake in tissues. PET scanning is commonly performed with simultaneous acquisition of CT and more recently with MRI for both functional and structural correlation.^28^ In patients with suspected PA sarcoma, PET will show high uptake allowing differentiation from chronic clot.^29,30^ However, in acute clot FDG, uptake may also be elevated, relative to unaffected vessels.^88^ In PAH, uptake within the lungs and right ventricle have been demonstrated^24–27^ although the clinical utility is uncertain.

### 1.4 Computed tomography

#### Summary statements

1. CT provides a non-invasive evaluation of vascular, cardiac, lung parenchymal, and mediastinal structures in patients with known or suspected PH.
2. Significant parenchymal abnormalities may be seen on CT evaluation in the presence of normal spirometry, particularly when there is a significant reduction in gas transfer factor.
3. CT pulmonary angiography to assess the pulmonary vasculature should be considered in patients presenting with PH.
4. Imaging biomarkers from CT in patients with suspected PH should include measurement of PA size, right-to-left ventricular ratio, and left atrial size.
5. CT aids the classification of PH.

CT is increasingly recognized as a valuable imaging modality for the evaluation of known or suspected PH. Advantages of CT include its widespread availability and accessibility, high spatial resolution, multi-planar imaging capabilities, and the ability to evaluate the pulmonary vasculature, lung parenchyma, cardiac, and mediastinal structures simultaneously.

#### CT evaluation of vessels

PA size can be easily measured and enlargement may suggest the diagnosis of PH. Routine measurement is recommended particularly in patients at risk of PH. For the diagnosis of PH in lung disease, a main PA diameter >29 mm had 84% sensitivity, 75% specificity, and 97% positive predictive value (PPV) for PH defined as a mPAP ≥ 25 mmHg.^89^ The reliability of measuring the main PA diameter and the ratio of the PA to aorta (Ao) ratio has also been studied in suspected PH. Investigators found that a PA:Ao ratio > 1 was 92% specific for a mPAP > 20 mmHg.^90^ Other reports also support the use of PA size in the clinical assessment of patients with PH.^91,92^ However, it has been shown that an increase in PA size also reflects disease duration and correlates only moderately with PAP.^93^ It has previously been postulated that the presence of interstitial lung disease independently influences PA size;^94^ however, in a large cohort of patients with suspected PH in association with interstitial lung disease, the presence and severity of interstitial lung disease did not influence PA size which was found to be a useful diagnostic marker in patients with and without interstitial lung disease.^95^

While contrast-enhanced CT angiography is the method of choice for the evaluation of suspected acute pulmonary embolism, its role in the evaluation of the pulmonary vasculature in the setting of CTEPH has been a more recent development. Multiple findings are associated with CTEPH, including intravascular organizing thrombi, webs, and regions of vascular narrowing or occlusion.^96^ Mosaic perfusion of the lung parenchyma and enlarged bronchial arteries are also commonly seen.^97–99^

Advanced CT capabilities have been studied in the evaluation of PH. DECT provides an assessment of relative perfusion and improves the detection of peripheral vascular occlusion. In one study,^85^ DECT showed 100% sensitivity on a per-patient basis compared to V/Q scintigraphy. However, there was imperfect agreement on a per-vessel basis. CT perfusion imaging may demonstrate residual perfusion abnormalities following therapy for acute pulmonary embolism, even in the absence of visualized thrombus on the angiographic portions of the study.^100^ Perfusion imaging can also estimate cardiac output and in a small pilot study could detect PH with high sensitivity and specificity.^101,102^

PAH is associated with vascular remodeling, including loss of arterial branching and increased vessel tortuosity. CT angiography can quantify these features, using fractal dimension and the ratio of actual vessel length to shortest linear distance to estimate tortuosity. Studies have shown these to correlate with hemodynamic measures in PAH.^103^ However, changes in the fractal dimension were found only in children with PAH, but not in adults.^104^ Loss of distal vascular volume has also been described in patients with severe emphysema^105^ and in patients with CTEPH.^106^

#### CT evaluation of lung parenchyma

CT is the gold standard for the evaluation of the lung parenchyma. In a large registry series, CT measures have proven useful clinically in the assessment of patients with PAH.^38^ Centrilobular ground-glass opacities are frequently seen in PAH and their presence on a CT performed for unexplained breathlessness should raise the possibility of this diagnosis.^38,107,108^ Features of cardiac decompensation, pleural effusion/septal lines, and inferior vena cava size predict outcome.^38^ The presence of emphysema or interstitial lung disease or bronchiectasis makes the diagnosis of PH in association with lung disease likely. The addition of expiratory imaging is helpful to assess for small airways disease. CT may also identify features associated with rare conditions such as pulmonary veno-occlusive disease (PVOD) and pulmonary capillary hemangiomatosis (PCH) (section 2.2). This is an important differential diagnosis to idiopathic PAH (IPAH) because PAH therapy may be indicated but has a much higher risk of severe adverse effects than IPAH.

#### CT evaluation of cardiac structure and function

CT has historically not been used for the evaluation of cardiac structural abnormalities as MRI and echocardiography are the current modalities of choice. Nonetheless, many cardiac findings associated with PH can be identified with non-gated contrast-enhanced CT, including enlargement of cardiac chambers, thickening of the RV free wall, and leftward deviation of the interventricular septum. CT can also identify structural abnormalities associated with congenital heart disease, such as partial anomalous pulmonary venous return and intracardiac shunts. Electrocardiogram-gated CT can be used to quantitatively assess RV and left ventricular (LV) function. Additionally, a decrease in distensibility of the main PA is highly correlated with the presence of PAH.^109–111^ A study has shown that dynamic contrast-enhanced CT can measure the transit of contrast, correlated with cardiac output.^102^ and is associated with the presence of PH.^101^

#### CT evaluation of mediastinal structures

CT provides detailed imaging within the mediastinum and may demonstrate findings that give information as to the etiology or severity of PH. Dilatation of bronchial arteries is a common finding in CTEPH, but less common in other forms of PH.^97,112^ A dilated esophagus in the setting of PH suggests the diagnosis of systemic sclerosis. Other mediastinal findings, while not specific to a given etiology, may suggest a poor prognosis. These include the presence of pericardial effusion, lymphadenopathy, and reflux of contrast into the hepatic veins.^38^

### 1.5 Magnetic resonance imaging

#### Summary statements

1. MRI enables comprehensive cardiac evaluation in patients with suspected PH.
2. MRI is the gold standard technique for the assessment of biventricular morphology and function and is highly suitable for monitoring patients with PH.
3. MRI provides prognostic value in PAH.
4. MRI aids the classification of PH particularly for left heart disease and chronic thromboembolic disease.

Cardiac MRI is the gold standard for quantification of RV volumes, mass, function, and flow hemodynamics in the pulmonary circulation.^113,114^ Cardiac MRI techniques allow for non-invasive assessment of RV function and structure using high spatiotemporal resolution imaging sequences with high accuracy and reproducibility without exposure to radiation.^113^ Furthermore, cardiac MRI can be used for assessment of myocardial tissue deformation properties (strain), global structural evaluation, and perfusion.^115–117^

#### Right ventricular size and function

RV hypertrophy and dilation reflect an increased afterload.^5^ In particular, in advanced stages of PH, a severely dilated and functionally compromised right ventricle has a negative effect on LV diastolic function by means of leftward septal shift and reduced LV filling associated with decreased RV stroke volume. Indeed, both RV and LV dimensional metrics have been shown to have diagnostic potential in treatment-naïve patients with PH and prognostic value in both adult and child populations.^118–120^ MRI-derived bi-ventricular functional and volumetric indices have been shown to have independent prognostic potential and differentiated incidental treatment-naïve and prevalent patients in a large group of patients (n = 576).^121^ Cine MRI derived indices including interventricular septal bowing, LV eccentricity, and ventricular dyssynchrony due to prolonged RV contraction time have been shown to correlate with invasive hemodynamics and are reflective of the overall hemodynamic condition and disease severity.^122,123^ Septal deviation measured by MRI is useful for the diagnosis of PH, but also in patients with left heart disease septal deviation > 160° can identify patients with elevated diastolic pulmonary gradient.^124^ In addition, MRI has proven useful in the diagnosis of PH in patients with chronic obstructive pulmonary disease (COPD), typically a challenging cohort for echocardiography.^125^

#### Late gadolinium enhancement and T1 mapping

Late gadolinium enhancement (LGE) imaging is used to identify focal myocardial pathology but has also been applied to investigate regional myocardial disease in the right ventricle as a response to elevated mechanical stress. The predominant focus has been on the RV free wall insertion sites to septum and how the extent of LGE corresponds to RV morphological and dynamic changes.^44,45,126–130^ Specifically, LGE was correlated with the reduced RV function, dilation, mass, and regionally specific LGE was also inversely associated with reduced longitudinal strain.^128,131^ Additionally, the presence of delayed enhancement at the RV insertion points has been associated with clinical worsening,^127^ though in a study using mortality as the endpoint, RV insertion point LGE was not of independent prognostic significance. Extension of the LGE into the interventricular septum was of prognostic significance at univariate analysis but was not significant at multivariate analysis.^45^ A limited number of studies have explored the role of coronary arterial flow in PH.^117,132^ Features may be seen in patients with PH with LGE imaging; however, its utility in the routine assessment of suspected PH is not proven. LGE imaging can be considered where intrinsic cardiac disease is suspected.

T1 mapping is a quantitative method of assessing myocardial health. Native T1 mapping is performed without the use of contrast agents and has been shown to be an excellent differentiator between a healthy and diseased myocardium.^133^ T1 has been shown to be elevated at the insertion points in PH in animal^134^ and human studies.^135–137^ T1 correlates with markers of RV remodeling^135^ and septal position;^138^ however, no clear diagnostic or prognostic role has been identified in PH.^138^

#### Right ventricular strain

Myocardial tissue deformation analysis has been assessed in patients with PH.^139–141^ RV morphology limits myocardial tagging and feature tracking to global longitudinal and circumferential deformation analysis. MRI-based feature tracking has been shown to have prognostic potential and was associated with the severity of PH.^142^ Measuring LV strain and torsion using tag MRI as part of ventricular interdependency and dyssynchrony investigations in CTEPH revealed left–right ventricular resynchronization post endarterectomy.^143^ However, the clinical utility of deformation analysis is yet to be determined.

#### Pulmonary artery and aortic flow measurements

Phase-contrast MRI (PC-MRI) enables assessment of flow waveform in major vessels and allows for accurate Q_p_:Q_s_ assessment, necessary in patients with suspected congenital lesions.^114^ Phase-contrast MRI of pulmonary flow is recommended for assessment of RV stroke volume due to variable tricuspid regurgitation and the challenges of contouring the right ventricle.^144^ Relative area change of the PA has been shown to be of clinical value,^145,146^ and recently has been shown to be independent of RV measurements and clinical data.^37^ Black blood slow flow has been shown to be a strong diagnostic marker as the flow characteristics of the main and branch vessels are visualized.^147,148^ Four-dimensional flow MRI (4D-Flow MRI) is an emerging technique allowing evaluation of flow, vorticity, and kinetic energy in any region of interest. Vortices have been noted in the main PA of patients with PH. The lifetime of the existence of a vortex has been shown to correlate with mPAP and may have utility in the identification of PH.^149–152^ 4D flow also has the additional benefit that it allows retrospective flow evaluation by selecting a 2D slice in any plane of the 4D dataset,^153^ whereas current techniques rely on the slice positioning at the time of the scan.

#### 3D MR perfusion and angiography

MR angiography (MRA) can show characteristic vessel patterns in subtypes of PH, including pruning in IPAH, thromboembolic obstruction and stenosis in CTEPH, and splayed vessels in COPD/emphysema.^113^ MRA is useful for the assessment of chronic embolus in the lobar and segmental PA vessels. Beyond the segmental level, assessment of the PAs with MRA is very challenging.^96^ In addition, a central embolus, particularly a wall adherent clot, can be missed if MRA is reviewed in isolation; standard white blood MRI sequences can assist with visualization of a central clot.^96^

Dynamic contrast-enhanced MRI perfusion is a promising technique for the assessment of chronic thromboembolic disease allowing visualization of pulmonary perfusion defects with sensitivity and specificity similar to that achieved with SPECT,^39^ with the advantages of higher spatial resolution and lack of ionizing radiation. Time-resolved MRA or dynamic contrast-enhanced (DCE) imaging can be used to measure passage of contrast bolus through the heart and lungs to assess pulmonary perfusion.^154,155^ This can be used to measure mean transit time, time to peak, and blood volume.^152,156,157^

### 1.6 Imaging in conjunction with invasive techniques

#### Summary statements

1. Catheter-based angiography is used primarily to assess patients with CTEPH considered as potential candidates for pulmonary endarterectomy or BPA.
2. Performance of catheter-based angiography requires skilled operators and should generally be performed in a PH referral center.
3. Non-invasive imaging approaches can be used to select patients for pulmonary endarterectomy.
4. Imaging measurements combined with catheter measurements may be used to study RV pressure and volume relationships.

#### Digital subtraction angiography

Catheter-based angiography involves rapid imaging of the PAs during the injection of contrast material through a catheter placed into the pulmonary arterial system.^158,159^ This used to be the primary method for evaluation of the pulmonary vasculature. However, given the development of CT and MR methods, these modalities can also be used.^96,106,160^ Catheter-based angiography may be used at expert institutions for the evaluation of chronic thromboembolism before pulmonary endarterectomy and is required for BPA.

#### Assessment of ventricular-arterial coupling

Imaging can be incorporated with invasive catheter-based methods for characterization of the mechanics of the right ventricle and the pulmonary arteries. Flow and volumetric MRI measurements in conjunction with pressures obtained from RHC can be used for assessment of ventricular-arterial coupling.^161–163^ One method for determining RV contractility involves computation of the pressure-volume loop while using balloon occlusion of the inferior vena cava, permitting a preload independent assessment of ventricular contractility.^164^ In practice, this method requires use of conductance catheters or the measurement of pressure and flow at the same time in order to construct the pressure volume loops with different degrees of preload modulated by the occlusion of the inferior vena cava. Conductance catheters typically require calibration of the volume signal from imaging, typically a baseline cardiac MRI. The “Single Beat” method using a cardiac catheter can be used to estimate Pmax. This has been used in conjunction with MRI to measure ventricular volumes and can serve as a surrogate for Ees/Ea,^165^ the relative utility of information from these methods remains an area of research.^166^ Cardiac MRI has been used to measure stroke volume and RV volumes and in conjunction with pressure measurements to construct pressure-volume loops and estimate RV contractility.^167–170^ An entirely MRI-based non-invasive method of measuring RV to PA coupling has been proposed, defined by RV stroke volume/RV end-systolic volume; however, this holds similar information to RVEF (right ventricular stroke volume / right ventricular end-diastolic volume).^171^ Studies have suggested added prognostic value of coupling measurements,^172,173^ although a recent large study suggested it did not add additional prognostic significance over RV volume alone in patients with PAH.^121^

## Section 2: Imaging adults with pulmonary hypertension

### 2.1 The accuracy of cross-sectional imaging to diagnose pulmonary hypertension and assess pulmonary hemodynamics

#### Summary statements

1. A number of CT and MRI findings are characteristic of PH.
2. Current qualitative approaches to imaging cannot be used to confidently exclude the presence of PH.
3. Quantitative data obtained from imaging can be used to diagnose PH and estimate pulmonary hemodynamics.

CT imaging is widely available and measurement of the PA size has been shown to correlate with mPAP;^174^ however, in established PH, there are progressive increases in PA size over time.^175^ Pulmonary artery enlargement may be seen in interstitial lung disease in the absence of PH,^94^ although a recent publication has shown equivalent diagnostic accuracy in patients with and without interstitial lung disease. In patients with systemic sclerosis in the absence of interstitial lung disease, a ratio of main PA to Ao diameter of at least 1 was highly predictive of the presence of PAH although a normal ratio did not exclude PAH.^176^

MRI is non-invasive, reproducible, and is considered the gold standard for assessing RV function.^177^ Studies have shown a high correlation between RV mass and ventricular mass index (VMI), the ratio of right-to-left ventricular mass, and mPAP pressure measured at cardiac catheterization.^178,179^ Recently investigators have shown that combining VMI and septal curvature improves the accuracy of estimating mPAP.^180^ By using MRI to calculate left atrial volume, pulmonary arterial wedge pressure (PAWP) can be estimated allowing calculation of the trans-pulmonary gradient.^180^ Cardiac output can be calculated from LV volumetric measurements or phase contrast of flow in the PA or Ao allowing an entirely non-invasive estimate of pulmonary vascular resistance (PVR) based on individually derived MRI measurements. Models using RVEF and average PA velocity have also demonstrated accuracy for estimating catheter-derived PVR.^181^ Studies comparing cardiac magnetic resonance cardiac MRI and RHC in patients suspected of PH have shown that an elevated VMI, reduced PA velocity, and the presence of increased gadolinium at the hinge points could predict the presence of PH with a positive predictive value of >0.9 although no cardiac MRI measure could confidently exclude PH.^179^ In summary, although able to estimate pulmonary hemodynamics and identify PH with high accuracy in certain groups, imaging is currently unable to exclude PH.

### 2.2 How helpful is imaging in identifying the cause of pulmonary hypertension and subtyping?

#### Summary statements

1. Different forms of PH and their subtypes may exhibit characteristic imaging features.
2. Echocardiography and MRI are useful for differentiation of pre and post capillary PH.
3. CT provides an accurate evaluation of lung structural abnormalities.
4. Nuclear medicine, CT and MR perfusion imaging can be used to exclude chronic thromboembolic disease.
5. Different combinations of imaging modalities can be employed tailored to local expertise and availability.

Many of the imaging findings associated with PH are common to most or all disease processes leading to PH. These findings may include enlargement of the central PAs, right-sided cardiac enlargement, and abnormalities of lung attenuation. Some imaging findings, however, are more specific and can help to distinguish among the various causes of PH. These are discussed in the following section.

#### Pulmonary arterial hypertension (group 1)

Within group 1 PAH, imaging findings may suggest a specific etiology. Patients with systemic sclerosis typically have a dilated esophagus, a central ground glass pattern, and often associated interstitial lung disease. Drug and toxin exposures may also be associated with mild parenchymal fibrosis or mosaic lung attenuation. In patients with portopulmonary hypertension, the presence of varices, features of liver cirrhosis, and splenomegaly are frequent. Anomalous pulmonary venous drainage should be sought as this is frequently associated with congenital heart disease, in particular a sinus venosus atrial septal defect which is difficult to detect by transthoracic echocardiography. An interatrial shunt may also be suggested by contrast visualized entering the left atrium via the interatrial septum. While enlargement of the PAs is common to all forms of PAH, it is greatest in patients with Eisenmenger physiology.^38^ Patients with Eisenmenger physiology may also have laminated proximal thrombus and calcification within the wall of the PA. Bronchial artery enlargement is most frequently observed in CTEPH but is also observed in patients with congenital heart disease^182^ and patients with IPAH and a BMPR-2 mutation.^183^ PVOD is rare and is characterized by mediastinal lymphadenopathy and interlobular septal thickening,^184,185^ with or without associated findings of alveolar edema, mediastinal lymphadenopathy in the setting of a normal sized left atrium. PCH is frequently associated with nodular foci of parenchymal infiltration. The triad of peripheral interlobular septal thickening, centrilobular ground-glass opacities, and mediastinal lymphadenopathy has a good association with PVOD and PCH.^184,186,187^

#### Pulmonary hypertension due to left heart disease (group 2), pulmonary hypertension due to lung diseases (group 3), and pulmonary hypertension with unclear and/or multifactorial mechanisms (group 5)

Within these groups, imaging may be useful to identify either left-sided cardiac enlargement (group 2) or diffuse lung disease (groups 3 or 5). Many lung diseases will have a distinctive radiographic appearance allowing for specific diagnosis and grading of severity. Characteristic structural features of patients with left heart disease which can be visualized on both CT and MRI include left atrial enlargement,^188–190^ absence of posterior displacement of the interventricular septum,^179^ and relatively normal RV volumes, although RV enlargement is seen in more severe disease particularly in the setting of severe tricuspid regurgitation. The presence of valvular and coronary artery calcification and evidence of previous cardiac surgery are more common in left heart disease although may also be present in patients with other forms of PH. Echocardiography and MRI allow a comprehensive functional assessment. Patients with left heart disease have less RV hypertrophy and compared to pre-capillary forms of PH have better preserved RV function. The absence of paradoxical septal motion/septal displacement in the setting of high right-sided pressures infers an increase in left-sided pressures. Echocardiography allows assessment of both systolic and diastolic dysfunction and the identification of valvular heart disease, in addition to identifying features suggestive of combined post- and pre-capillary disease.^191^

#### Chronic thromboembolic pulmonary hypertension (CTEPH) and other pulmonary artery obstruction (group 4)

A large prospective study estimated the risk of developing CTEPH after a pulmonary embolism at 3.8% at two years.^192^ Imaging plays a critical role in the evaluation of suspected CTEPH, although the exact role of each imaging modality is debated. Some of this uncertainty reflects the rapid development of imaging technologies. Chest radiographs may suggest the diagnosis of CTEPH – with cardiomegaly, asymmetrical pulmonary artery enlargement, pruning of the vasculature and subpleural scarring; however, they are not diagnostic. Historically decisions on diagnosis and surgical management were based on V/Q scintigraphy and conventional pulmonary angiography with RHC. The role of V/Q scintigraphy (either planar or SPECT imaging) has changed. SPECT is recommended over planar imaging as it has higher diagnostic accuracy.^193^

The current ESC/ERS guidelines recommend that V/Q scintigraphy be performed in all patients with suspected CTEPH.^194^ These recommendations for V/Q scanning are in part based on clinical experience, previous recommendations, and on older data.^195–197^ These data demonstrated a significant improvement in the detection of CTEPH with scintigraphy compared to CTPA. However, rapid developments in CT technology have led to marked improvement in disease detection and characterization. More recent studies in experienced centers demonstrate that CTPA has high diagnostic accuracy for CTEPH.^39^ Key imaging findings include identification of eccentric thrombus, intravascular webs, stenoses with or without post-stenotic dilatation, and occlusions. Bronchial artery dilatation (commonly described as a diameter of >2 mm) is more commonly seen than in other forms of PH. The presence of bronchial artery dilatation is associated with a better outcome following pulmonary endarterectomy. A mosaic perfusion pattern is seen in the vast majority of patients with CTEPH. Its presence should alert the observer to the possibility of CTEPH but should be differentiated from the mosaic pattern seen in small airways disease where often single or small clusters of lobules are involved in contrast to larger geographical areas typically seen in CTEPH. Performance of expiratory CT may be helpful in this setting. Peripheral areas of sub-pleural scarring and cavitation representing healed infarcts may also be seen in approximately 10% of patients with CTEPH.^198^ DECT imaging generates maps of regional iodine density in the lung parenchyma as a surrogate for perfusion. This may further improve the evaluation of suspected CTEPH by better demonstrating regions of decreased or absent blood flow^199^ and has been shown to have excellent agreement with SPECT.^200^ Although the availability of this technology is relatively limited, iodine mapping using CTPA with an unenhanced pre-scan is an emerging technique, which generates a lung perfusion map. In contrast to DECT, it does not require specialized hardware but involves subtraction of unenhanced images from the contrast-enhanced study. This has the advantage that subtle abnormalities that may be missed on the angiography are unlikely to be overlooked on a perfusion map thus improving detection of subtle webs and distal disease.^81^

By providing an assessment of the lung parenchyma and the mediastinum CT can be helpful in identifying the presence of diffuse lung diseases and emphysema (groups 3 and 5) and excluding pulmonary vascular obstruction from a central mass. Parenchymal evaluation may also be valuable in identifying features suggestive of interstitial edema, PVOD, or vasculopathy though there is overlap in the imaging features. CT may also identify features suggestive of large vessel vasculitis or PA sarcoma which may not be readily appreciated on projectional angiography.

MRI can also provide an evaluation of the pulmonary vasculature and is increasingly being used in select centers for the evaluation of known or suspected CTEPH. 4D DCE lung perfusion MRI techniques^201^ are widely available on current MRI systems and have shown excellent test performance in diagnosing CTEPH in a single center registry setting.^39^ In addition, a non-contrast, free-breathing ventilation perfusion MRI technique, known as the Fourier Decomposition (FD)-MRI method^202,203^ has recently shown initial encouraging results in diagnosing chronic pulmonary embolism;^204^ however, this technique needs to be confirmed in larger multicenter studies. Combined cardiac MRI and time-resolved MRA exam is suitable for detailed treatment response evaluation before and after pulmonary endarterectomy as well as BPA in CTEPH patients.^205,206^

CTPA has largely replaced invasive catheter-based angiography as the initial morphological test for PA evaluation in most centers. However, catheter-based angiography may still have a role in the evaluation of CTEPH. Older data suggest that catheter-based angiography may provide a better assessment of the extent of pulmonary vascular obstruction than V/Q scintigraphy.^207^ Additionally, some expert centers prefer catheter-based angiography for evaluation of the extent of thrombotic disease before PEA.^208^ Again, CT performance relative to catheter-based angiography is generally poorer in older studies^159^ compared to more recent data.^209,210^ This difference again likely reflects significant advances in CT technique and technology as well as greater awareness of CTEPH and its imaging features in imaging circles. When there is diagnostic uncertainty regarding the extent and distribution of chronic thromboembolic changes on the basis of a single imaging modality (usually CT), the use of a second morphologic imaging modality (MRA or catheter-based angiography) may prove complementary by improving confidence in subtle lesions or identifying others. This can augment surgical decision-making.

In recent years, BPA has emerged as an effective therapeutic modality in selected patients with CTEPH. This has put greater emphasis on the evaluation of distal segmental and subsegmental vasculature. It is generally considered that at the subsegmental level, catheter-based angiography outperforms non-invasive morphological techniques (CT/MRI) which has resulted in increased utilization. Clinical criteria and imaging algorithms for the selection of patients for BPA vary between centers but catheter-based diagnostic angiography for potentially suitable candidates permits confirmation of suitable extent and distribution of disease as well as the patients ability to tolerate a BPA procedure (ability to maintain breath-hold and tolerate the required period on the catheter table).^211^

### 2.3 Echocardiography and cardiac MR: what are the advantages and disadvantages of each modality?

#### Summary statements

1. Echocardiography is more widely available, lower in cost, and more portable than MRI.
2. Echocardiography is superior to MRI for the evaluation of valvular heart disease and is more established in the assessment of diastolic function.
3. MRI provides more accurate quantitative assessment of RV morphology and function.
4. MRI is more suited to serial assessment than echocardiography due to higher reproducibility.

Echocardiography and cardiac MRI provide value in the assessment of patients with PH. Echocardiography is well established in the initial assessment of patients with suspected PH. It has also been evaluated in the serial assessment of patients with PH^66,67^ and has been found to be prognostic and is recommended in current guidelines at follow-up and following treatment change.

#### Technical factors

Echocardiography has high temporal resolution, is widely available, low in cost, portable, and less affected by arrhythmia than MRI although real-time imaging^212^ has helped to counter this limitation at the cost of lower spatial resolution. Echocardiography is more operator-dependent and is less reproducible. MRI has the advantage that it has better contrast resolution and is able to image in any plane making it more suited to accurately quantify RV morphology and function. Furthermore, contrast imaging allows assessment of focal abnormalities of the myocardium and an assessment of myocardial perfusion.^113,116^

#### Estimation of pulmonary hemodynamics

In a systematic review of the literature, the sensitivity and specificity for echocardiography for diagnosing PH was 83% (95% confidence interval [CI] = 73–90) and 72% (95% CI = 53–85), respectively. However, echocardiography is less accurate, in lung disease with sensitivity of 60% and specificity of 74%.^213^ Empiric approaches have been used to estimate pulmonary hemodynamics using MRI.^178,180^ In a recent study, mPAP was accurately estimated using multivariate regression analysis of MRI indices, with ventricular mass index and interventricular septal angle having additive value in model for estimation of mPAP.^180^ It has recently been shown that with the inclusion of black blood pulmonary arterial slow flow in addition to ventricular mass index and interventricular septal angle high diagnostic accuracy can be improved further.^147^

#### Right ventricular volumetric and functional assessment

Complete visualization of the right ventricle with echocardiography can be challenging, particularly in lung disease, preventing a complete volumetric evaluation of the right ventricle; this may be partially negated by the use of 3D echocardiography. MRI has the advantage of complete volumetric evaluation of the cardiac chambers with good reproducibility of volume, mass, and ejection fraction^214^ and is considered the gold standard for serial assessment of RV volume and function.^215^ Inaccuracies in segmentation of the right ventricle at the base of the heart and segmentation of ventricular trabeculations may be improved using threshold-based approaches.

Echocardiography, using pulsed wave and tissue Doppler, is an established technique for the assessment of diastolic function. MRI phase contrast evaluation of the mitral annulus is feasible^216^ and in a small study imaging of the mitral valve annulus, in combination with mitral valve and pulmonary venous flow, was as accurate as echocardiography.^217^ Other techniques such as myocardial SPAMM tagging^218^ and 4D three-directional velocity encoded MRI^219^ have been used to evaluate LV diastolic function. Long analysis times may reduce the applicability for routine clinical use.

#### Valvular heart disease

PH may occur in the setting of valvular heart disease. Echocardiography is the most useful non-invasive technique and has the advantage over MRI in that it has high temporal resolution and allows accurate quantification of the severity of valvular heart disease. Left-sided valvular heart disease is a common cause of group 2 PH. Contemporary studies suggest a prevalence of PH of 30–40% in patients with mitral stenosis^220^ upwards of 30% in patients with severe mitral regurgitation;^221^ in asymptomatic patients, the presence of PH serves as an indication for valve surgery.^222^ With the emergence of transcatheter aortic valve replacement, PH has been noted in up to 75% of patients with severe aortic stenosis.^223^ Currently, echocardiography is the recommended non-invasive technique for the assessment of the presence and severity of valvular disease given its advantages over MRI including availability, low cost, high temporal resolution, and widespread experience over many years. Despite this, there are instances in which echo is limited due to poor acoustic windows, lack of agreement across quantitative methods, and significant inter- and intra-observer variability, which has led to interest in the emerging role of MRI in the assessment of valvular heart disease.^224,225^

### 2.4 Can imaging replace cardiac catheterization in the assessment of suspected pulmonary hypertension?

#### Summary statements

1. Cardiac catheterization is the gold standard for the measurement of PAP.
2. CT and MRI may suggest the diagnosis of PH and quantitative MRI metrics can be used to confirm the presence of PH with high accuracy.
3. At diagnosis MRI and cardiac catheterization provide equivalent levels of prognostic information.
4. Cardiac catheterization is currently the only way to identify patients with IPAH likely to benefit from calcium antagonist therapy.

Current ESC/ERS guidelines^226^ recommend RHC for the definitive diagnosis of PH, assessment of intra-cardiac shunting, and vasodilator testing in selected patients to identify the 10% of patients with IPAH who may benefit from high-dose calcium antagonist therapy. Measurements such as right atrial pressure, cardiac index, and mixed venous oxygen saturation have prognostic value,^227–229^ and serial measurements are currently recommended to assess the response to therapy.^226^ The procedure requires meticulous attention to detail but in expert hands is safe in adults with a morbidity of approximately 1% and mortality of 0.055%,^230^ although the risk of complications is significantly higher in children. Well established criteria at right heart catheter exist to assess patients with IPAH likely to benefit from calcium antagonist therapy. However, no such criteria exist for imaging metrics.

Although current guidelines discuss the role of CT and MRI in the classification of PH, they are currently considered an adjunct and not yet considered a replacement for RHC.

### 2.5 What is the role of imaging in assessing prognosis and response to treatment?

#### Summary statements

1. Echocardiography allows assessment of RV function and measurements such as right atrial area, RV fractional area change, and tricuspid annular plane systolic excursion have prognostic value.
2. CT imaging provides prognostic information in PAH but its role in follow-up is currently limited by exposure to radiation.
3. A number of cardiac MRI metrics have prognostic value and changes in cardiac MRI parameters at follow-up reflect changes in functional capacity and survival.
4. Adjustment of RV functional measurements for age and sex improves prognostication.
5. Changes in RV function measured by MRI have prognostic value.

Echocardiography is widely available and a number of metrics have been shown to have prognostic value including right atrial area,^231^ RV fractional area change, TAPSE,^232–234^ and the presence of a pericardial effusion.^231,235^ Echocardiography is widely used, in many centers to assess the response to treatment, acknowledging issues of operator-dependency and reproducibility.

Several measurements made at CTPA—including RV to LV ratio, right atrial size, and a posteriorly deviated inter-ventricular septum—predict prognosis in PAH subgroups while the presence of pleural effusions, septal lines, and increased inferior vena cava area were independent predictors of a worse outcome in PAH at presentation regardless of subgroup.^38^ Although this may be helpful at presentation to guide urgent assessment and introduction of emergency therapies, the availability of other imaging modalities and associated radiation exposure necessitates that CT is not recommended in the assessment of treatment response.

In contrast, the highly reproducible nature and non-invasive and non-ionizing nature of MRI makes it an ideal modality to assess treatment response and in the EURO-MR study^236^ changes in MRI measured cardiac index and RV RVEF correlated with changes in World Health Organization functional class and survival. A study examining changes in cardiac MRI parameters demonstrated that this was a better predictor of outcome than PVR measured invasively at cardiac catheterization.^215^ MRI metrics including increased RV volumes, reduced LV volumes, stroke volume, cardiac output, and pulsatility of the vasculature predict a worse outcome in PAH although there is currently no large study comparing the prognostic value of cardiac MRI and right heart catheter measures.

### 2.6 How can we improve imaging techniques to make them more acceptable to patients?

#### Summary statements

1. Minimizing radiation dose by the use of non-ionizing techniques where possible and implementation of dose reduction protocols will reduce the risks to patients.
2. Involvement of patients in investigative decision-making will allow a more tailored approach to investigation.

While plain chest radiographs and echocardiography are generally well-tolerated and acceptable examinations, cross-sectional techniques do have some specific limitations and issues, in particular radiation doses for CT and claustrophobia for MRI.

The diagnostic information provided by CT needs to be balanced with the radiation dose. In a life-shortening illness, concerns regarding radiation exposure rarely impact on the decision to perform CT imaging at the time of diagnosis. Advances in dynamic imaging, with improved temporal resolution, has provided hope that gated CT can challenge MRI in the assessment of cardiac function; however, such examinations typically involve higher doses of radiation. Using iterative reconstruction, dose reduction can be achieved.^237^ In fact, large reductions in dose to less than one-third the dose of standard acquisitions have been achieved without loss of image quality using iterative reconstruction,^238^ and the use of dynamic Z axis collimation has been shown to further reduce dose.^239^

Limitations of MRI include long scanning times and scanner noise, and frequently patients feel claustrophobic (5%) leading to incomplete examinations. Developments to reduce scanning time, e.g. novel rapid imaging techniques, and limiting the study to the most clinically relevant sequences may help. The more widespread installation of wide bore scanners may help to reduce claustrophobia and make MRI a more acceptable imaging modality for all patients. The use of media entertainment may improve acceptability to patients. More patient involvement in discussions around the implications of the tests are needed. Patient participation is advised to determine the issues most relevant to patients to help develop imaging services.

## Section 3 Imaging pathway for suspected pulmonary hypertension in adults

### 3.1 Current guidelines

The ESC/ERS guideline on diagnostics and therapy of PH review current imaging modalities and make a number of recommendations for incorporation in a diagnostic strategy.^2^ Echocardiography is recommended as a first-line non-invasive diagnostic investigation in case of suspicion of PH. Chest X-ray and high-resolution CT are recommended in patients with high or intermediate probability of PH following echocardiography and high-resolution CT should be considered in all patients with PH. Ventilation/perfusion or perfusion lung scan is recommended in patients with unexplained PH to exclude CTEPH. Contrast CT angiography of the PA circulation is recommended and pulmonary (catheter-based) angiography should be considered in the work-up of patients with CTEPH. There are no recommendations for the use of MRI as part of the diagnostic strategy or algorithm and no discussion of emerging techniques.^240,241^ Current American College of Chest Physicians guidelines on PH provide no specific recommendations on employing imaging as part of a diagnostic strategy in patients with suspected PH.^242^

The Cologne Consensus Conference 2016 provides no additional imaging guidelines.

### 3.2 PVRI diagnostic imaging pathway

#### Initial assessment and identification of risk factors

**Fig. 1.**
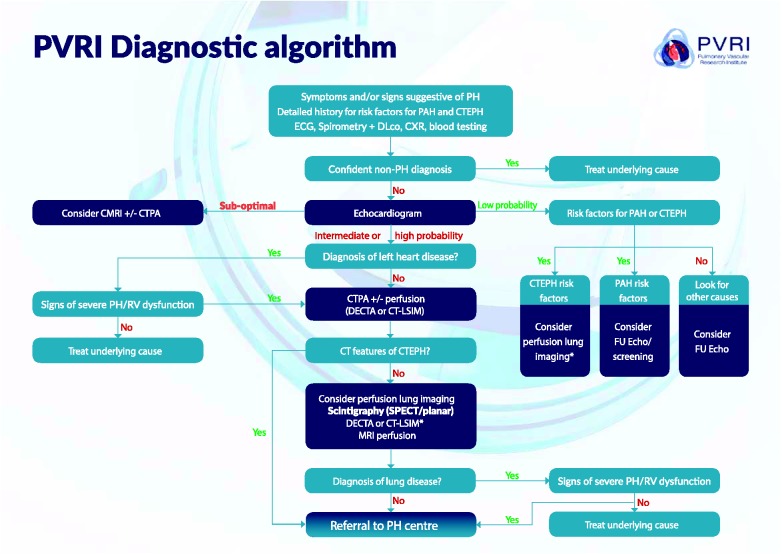
PVRI diagnostic algorithm. In this algorithm, patients are classified into low, intermediate, and high risk of PH according to ESC/ERS guidelines.^2^ See section 3.2 for a detailed description of how to navigate the algorithm. *For patients with rapidly progressive symptoms and a high probability of PH on echocardiography do not delay referral to PH centre to complete imaging investigations. PH, pulmonary hypertension; PAH, pulmonary arterial hypertension; CTEPH, chronic thromboembolic pulmonary hypertension; ECG, electrocardiogram; DLco, diffusing capacity of the lungs for carbon monoxide; CXR, chest radiograph; CMRI, cardiac magnetic resonance imaging; CTPA, computed tomography pulmonary angiography; DECTA, dual-energy computed tomography angiography; CT-LISM, computed tomography lung iodine subtraction mapping; SPECT, single photon emission computed tomography; RV, right ventricular.

In patients presenting with symptoms and/or signs suggestive of PH, a detailed history and the results of basic tests are key in determining the diagnostic strategy (Fig. 1). Risk factors for treatable forms of PH (PAH and CTEPH) must be sought as their presence reduces the threshold for further imaging. Basic investigations including an electrocardiogram, lung function with gas transfer factor, and a plain radiography may suggest an alternative diagnosis^194^ or increase the probability of PH. Further investigation may not be required when a confident alternative diagnosis can be made.

#### Echocardiograpy

Echocardiography is the recommended first-line imaging modality in the assessment of suspected PH and allows evaluation of cardiac structure and function and an estimate of PAP. Following echocardiography, patients should be stratified into those at low, high, or intermediate probability of PH according to ESC/ERS guidelines.^194^ **Where patients have rapidly progressive symptoms and a high probability of PH from echocardiography, physicians should not delay referral to expert centers until the above investigations are completed.**

#### Sub-optimal echocardiography

For patients with a sub-optimal echocardiogram, imaging with cardiac MRI can be used to identify patients at increased risk of PH although currently used metrics cannot confidently exclude mild PH.^147^ A number of metrics on CTPA have been shown to reflect elevated PAPs in addition to providing information on other potential causes for breathlessness; although evidence is limited, this may be considered in selected patients.^91,95^

#### Low probability of pulmonary hypertension from echocardiography

For symptomatic patients identified as low probability from echocardiography, further assessment is dependent on the presence or absence of risk factors. For those with risk factors for CTEPH, perfusion lung imaging is recommended using CT imaging (CT-LSIM of DECTA), nuclear medicine techniques (ideally SPECT), or MRI perfusion imaging.^39,81,86,243^ If risk factors for PAH exist, the diagnostic strategy will be dependent on the risk factor; in systemic sclerosis, given the high prevalence of PAH in symptomatic patients, further evaluation is advised and a number of screening regimens exist such as DETECT.^244^ For other at-risk patients, an interval echocardiographic examination may be appropriate.^194^

#### High or intermediate probability of pulmonary hypertension from echocardiography and assessment for left heart disease

For patients with intermediate or high probability of PH, the echocardiogram should be evaluated for evidence of left heart disease such as significant valvular heart disease, LV systolic or diastolic dysfunction. If present, the history should be re-reviewed to assess for risk factors for left heart disease (hypertension, obesity, coronary artery disease, diabetes mellitus, atrial fibrillation). Where risk factors for PAH or CTEPH are absent and risk factors for left heart disease present, PAP modestly elevated, left atrial size increased, no paradoxical septal motion, and/or significant RV dysfunction present, then no further investigation to assess for PH may be required. However, where risk factors for PAH or CTEPH are present, RV function is severely impaired, systolic PAP is severely elevated (≥70 mmHg), and/or paradoxical septal motion exists, then further investigation to exclude other causes of PH should be considered.^26^ If no features of left heart disease exist, patients should undergo CT pulmonary angiography if no contraindications exist.

#### CT pulmonary angiography

Where left heart disease is excluded, or if present and other causes of PH cannot be confidently excluded, then cross-sectional imaging with CT including CT pulmonary angiography should be considered as it can aid in: (1) assessment of the likelihood of PH; (2) classification of disease (identifying features of co-existing lung disease or left heart disease); and (3) identification of patients with CTEPH.^38^ If features of CTEPH are identified at this stage, patients should be referred directly to a center experienced in the management of PH for further evaluation.

#### Perfusion lung imaging

If CTPA is sub-optimal, indeterminate, or is performed by a center not experienced in the assessment of PH and CTEPH is not identified, in the absence of significant parenchymal lung disease, perfusion lung imaging (Q-SPECT of 3D MR perfusion) is advised at this stage. CT lung subtraction iodine mapping (CT-LISM) or DECT in addition to directly visualizing abnormalities in the pulmonary arterial tree also allows construction of perfusion lung maps, preventing the need for other forms of perfusion lung imaging to exclude CTEPH.^81,86,199,245^

Supplementary investigations including tests to assess for conditions associated with PAH such as connective tissue disease and HIV infection should be considered.^1,194^

#### Review and integration of imaging investigations with other tests and assessment for respiratory disease

Following imaging, the results should be integrated with the patient’s clinical characteristics. CT imaging may identify unexpected findings such as thromboembolic disease or parenchymal lung disease. Given the current lack of evidence for specific interventions targeting the pulmonary vasculature for patients with PH in the context of respiratory disease current therapies should be aimed at the underlying condition, recognizing that the presence of PH identifies patients at increased risk of death; where appropriate options such as transplantation should be explored. In patients with respiratory disease with risk factors for PAH or CTEPH, significant RV dysfunction or severe elevation in systolic PAP (≥70 mmHg), referral to a PH center should be considered; selected patients may be entered into studies or receive a trial of therapy. In addition to pulmonary vascular phentoypes increasingly recognized in respiratory disease,^4,246,247^ these patients may have other forms of PH such as undiagnosed connective tissue disease or CTEPH.^247^ Where uncertainty exists, discussion or referral to a PH center is recommended.

#### Referral or discussion with a pulmonary hypertension referral center

PH referral centers provide an environment where specialists are experienced in the assessment of patients with suspected PH. They also provide specific therapies and support for people affected by PH. Imaging investigations will be reviewed and, where sub-optimal, may be repeated. At this stage, further investigation will usually be dependent on the pre-test probability of different forms of PH (Table 1). For patients considered for treatment, cardiac catheterization is recommended.

**Table 1.** Recommended imaging investigations in adults with pulmonary hypertension considered for specific pulmonary vascular interventions.

	Imaging investigations
All patients	Echocardiography
	CTPA or DECTA or CT-LSIM
	Perfusion lung imaging in selected patients (see section 3.2)
Liver disease suspected or known	Ultrasound scan of liver with portal Doppler
Congenital heart disease suspected or known	Consider:
	MRI with Qp:Qs
	Transesophageal echocardiogram
Left heart disease or respiratory disease	Cardiac MRI or further echocardiographic studies to assess RV function may reduce the need to proceed to cardiac catheterization
Suspected CTEPH	Consider: MR angiography or digital subtraction angiography
	Lung ventilation
	If malignant obstruction suspected FDG-PET CT recommended
Unexplained hypoxemia	Consider: Bubble echocardiography
	Renal Q SPECT
	Cardiac MRI Qp:Qs
	MR time-resolved imaging
Follow-up of RV function	Echocardiography
	Cardiac MRI

CTPA, CT pulmonary angiography; DECTA, dual-energy computed tomography angiography; CT-LSIM, computed tomography lung subtraction iodine mapping; CMRI, cardiac magnetic resonance imaging; Qp:Qs, pulmonary–systemic flow ratio; FDG-PET CT, fluorodeoxyglucose-positron emission tomography computed tomography; SPECT, single photon emission computed tomography.

#### CTEPH suspected

For those with evidence of CTEPH on CTPA or with risk factors such as previous pulmonary embolus, deep venous thrombosis, splenectomy, or pacemakers, further evaluation of (1) the pulmonary vasculature with DSA or MRA, (2) lung perfusion, with SPECT or 3D MR perfusion, DECTA/CT-LISM, (3) lung ventilation, (4) biventricular function, with cardiac MRI or 3D echo, and (5) coronary or further cardiac valvular assessment, if risk factors for ischemic heart disease exist or co-existant valvular heart disease is noted, and the patient is considered a candidate for pulmonary endarterectomy, may be performed. The choice of investigations is also dependent on the preference of PH referral center, where pulmonary endarterectomy or BPA, is being considered. RHC with measurement of PAWP and PVR will aid decisions regarding appropriateness of the intervention and to confirm the presence of PH. Where filling defects extend into the proximal PA and or RV outflow tract, other conditions such as sarcoma should be considered and FDG-PET-CT may be helpful.^248,249^

#### Patients with risk factors for specific forms of pulmonary hypertension

In patients with risk factors for specific forms of PAH, further investigation should be tailored; ultrasound examination of the liver with portal Doppler ultrasound should be performed in patients suspected of underlying liver disease/portal hypertension. In cases where congenital heart disease is suspected (features such as anomalous pulmonary venous drainage or atrial septal defect may have been detected on CT or transthoracic echocardiography), transesophageal echocardiography and or cardiac MRI to estimate Qp:Qs ratio and cardiac catheterization with a saturation run should be considered.^250,251^ In patients with PH, where the cause is felt to primarily related to left heart disease or respiratory disease, RHC may be required to assess disease severity particularly if a trial of treatment is contemplated. Further assessment of RV function in these patients may be helpful, particularly where the echocardiographic assessment of RV function was challenging; findings at cardiac MRI of preserved or mildly impaired RV function or a normal septal angle in PH-LHD (suggesting the absence of a pre-capillary component) may negate the need for RHC.^124^

#### Unexplained pulmonary hypertension

Where no obvious cause of PH exists, following review of imaging and integration with other clinical characteristics, cardiac catheterization with vasodilator testing should be performed to identify the 10% of patients with IPAH who have a fall in mPAP or at least 10 mmHg to <40 mmHG with no reduction in cardiac output, who may respond to treatment with high-dose calcium channel blockers.^194^

#### Unexplained hypoxemia

Hypoxemia in PH is uncommon at the time of diagnosis in the absence of respiratory disease, a right-to-left shunt, or a severely reduced gas transfer factor. If a right-to-left shunt is suspected, a bubble echocardiogram, renal perfusion SPECT, or MRI (time-resolved imaging or Qp:Qs) should be considered.

#### Monitoring of patients at follow-up

Following diagnosis, follow-up assessments of RV function are recommended to aid risk stratification^119,215^ in combination with a clinical assessment and a measurement of exercise capacity. Cardiac MRI or echocardiography can be used to assess RV function. In selected cases, follow-up cardiac catheterization may be performed.

## Section 4.0: Imaging children with suspected pulmonary hypertension

### Summary statements

1. Echocardiography is recommended as the initial imaging investigation in children with suspected PH.
2. Performance of cardiac catheterization in children frequently requires general anesthesia and is associated with a higher risk of complications than in the adult population.
3. Diagnostic imaging strategies differ in children compared to adults reflecting significant differences in disease etiology.
4. MRI is of additional value in the initial evaluation and follow-up of PH in conjunction with other non-invasive techniques such as echocardiography.
5. Imaging techniques should be modified where possible to provide adequate diagnostic information while avoiding anesthesia.

### 4.1 Introduction

Imaging provides valuable information that aids the management of patients with PH, from diagnosis and accurate phenotyping, through to monitoring disease and assessing response to therapy. The majority of imaging modalities in medicine and their implementation in hospital environments have been developed with the needs of adult patients in mind. While the underlying philosophy and principles of imaging in children are the same as in adult PH, there are some challenges which may be more pertinent to imaging children. With appropriate modification these can be overcome.

#### 4.2 Differences in the spectrum of disease between adults and children

While pulmonary vascular disease pathophysiology is very similar, the context in which it occurs in children is often very different to adult cohorts of PH. Most notably, children are much more likely to have PH in the context of congenital or developmental abnormalities, whereas in adult populations co-morbid diseases of aging may be more important. Large cohorts of pediatric patients with PH demonstrate that PH related to congenital heart disease and PH related to developmental lung disorders predominate with IPAH responsible for approximately 20% of published cohorts and CTEPH responsible for <1% of cases.^252^ Furthermore, approximately 30% of children with PH have more than one potentially causal association.^253^ Finally, in a large proportion of children with PH, the PH is associated with other rare conditions. Taken together, this means that the pre-test probabilities of different PH etiologies differ from that in adults. This, in turn, affects the overall diagnostic imaging strategy. A diagnostic algorithm for children has recently been published following the World Symposium of Pulmonary Hypertension and reflects differences between adults and children.^254^

#### 4.3 Scale

The pediatric period covers a period from birth to adulthood. The body undergoes enormous growth and development during this period of time and body size can increase by almost two orders of magnitude. Organ structure, function, maturity, and complexity continue to develop through childhood and again enormously during puberty, profoundly affecting physiology.

The most obvious change through childhood is in size. This produces challenges when aiming to distinguish normal organ size from abnormal, e.g. ventricular volume. A number of approaches have been adopted to address this challenge. The first is to establish normative data for children throughout childhood and express these in terms of centile charts or standard deviation (Z) scores. Normative values in echocardiography in large populations of healthy children are increasing with normative echocardiographic values published in pediatrics with Boston z-scores.^255,256^ Normative values in CMR in large populations of healthy children is challenging and normative data are sometimes lacking. A second approach is to adopt ratio–metric relationships, i.e. the parameter in question is simply divided by a measure of body size, e.g. body surface area (BSA), or is expressed as a ratio against another cardiovascular parameter in the same patient, e.g. PA to Ao size ratio; however, these approaches have significant limitations. A more appropriate and physiologically sound approach to scaling may be to adopt allometric scaling relationships. This approach divides the cardiovascular variable of interest by the body size variable raised to a scalar exponent in the form x/y^b^. There are data across a huge range of scales and species which show empirically that this approach eliminates the effect of body size on cardiovascular structure and function. This approach to scaling or normalization has not been widely adopted and therefore the relevant scaling exponents are not well established or accepted.

#### The effect of scale on imaging resolution

By definition, in children spatial scales of structures are smaller (i.e. children are smaller) and temporal scales are typically shorter (i.e. children have higher respiratory rates and higher heart rates). This therefore affects imaging quality at any given spatiotemporal resolution. While some adaptations are possible for example higher frequency ultrasound probes for echocardiography, other imaging modalities suffer from fundamental physical and engineering limits to their spatial temporal resolution. Table 2 suggests some approaches which may improve spatiotemporal resolution of imaging modality such that they are suitable for smaller patients.

**Table 2.** Imaging modalities used in pediatric pulmonary hypertension.

Modality	Adaptation	Problem
Echocardiography	Higher frequency probes provide better spatial and temporal resolution in children; optimized sector width and focal length improves image quality in children	High heart rate, small structures but better echo windows reduced distance from probe to structure of interest
CT imaging	Multislice	Small structures, high heart rates, movement, difficulty in breath holding, need to avoid sedation anesthesia
MRI	Rectangular field of view, partial Fourier encoding, patient-friendly MRI environment, use of novel real-time sequences/under-sampling with novel reconstruction	Small structures, high heart rates and respiratory rates, movement and difficulty breath-holding, need to avoid anesthesia

CT, computed tomography; MRI, magnetic resonance imaging.

### 4.4 Intellectual/emotional maturity

Many imaging modalities require the cooperation of the patient to achieve optimal imaging results. Children are often emotionally and intellectually less mature than adults; thus, securing their cooperation can be more challenging and time-consuming. One approach to this which is widely used is to sedate or anesthetize children; however, sedation and anesthesia in children with PH is associated with a substantial risk of morbidity and mortality and it is desirable to avoid this wherever possible. Imaging in an appropriate environment with adequate time and support, e.g. play therapist involvement and time to familiarize patients with the environment, can substantially improve cooperation and imaging quality. Distraction techniques and allowing parents into the imaging room are extremely helpful.

### 4.5 Echocardiography in pediatric pulmonary hypertension

Echocardiography is used as the initial screening diagnostic imaging for the diagnosis of pediatric PH and the most important non-invasive tool that is used for routine assessment.^47,48,252,257–259^ It is used for continued follow-up and medication management.^260^ Developments in echocardiography in the past two decades have led to new insights into the structure and function of the right ventricle and its role in various diseases including PH.^261,262^ Conventional imaging includes assessment of anatomy in two dimensions (2D), hemodynamics via Doppler echocardiography, and qualitative and quantitative evaluation of RV and LV function.^47,48,258,260,263–265^ Advanced echocardiography includes evaluation of right heart size and function, myocardial mechanics, and estimated RV to PA coupling ratio.^266–270^ Table 3 shows the advantages and limitations of each of echocardiographic techniques used in pediatric PH. In recent years, different echocardiography parameters have been found in small studies to be useful in identifying high-risk patients who are likely to develop adverse clinical outcomes.^269,271–274^ Table 4 demonstrates the echocardiographic views needed to obtain the functional parameters in pediatric PH.

**Table 3.** Echocardiographic variables used at diagnosis and follow-up in pediatric PH.

Echo variables	Interpretation	Advantage	Limitations	References
Interventricular septal flattening	Mild, moderate, or severe to indicate the severity of PH	Quick visual assessments when there is not adequate TR peak velocity to estimate RVSP	Qualitative. Can occur in systole and diastole depending on pressure or volume overload	^317,318^
			Poor validation with clinically relevant measures	
TR peak velocity (m/s)	RVSP = SPAP = 4(TR max)^2 ^+ mRAP. If TR velocity is >3 m/s, PH may be suspected	Easy to obtain	75% is measurable	^319^
			Modest correlation with SPAP	
mPAP (mmHg)	mPAP = 4 V(early peak pulmonary regurgitation velocity)^2 ^+ RAP	Alignment of PR maybe better than TR	PR required for measurement	^260,263^
			Modest correlation with mPAP	
			Not substitute for cardiac catheterization	
Diastolic PAP (mmHg)	DPAP = 4 V(end-diastolic pulmonary regurgitation velocity)^2 ^+ RAP	Alignment of PR maybe better than TR	PR required for measurement	^260,263^
TAPSE (mm)	Longitudinal systolic function	Easy to obtain, impaired RV systolic function when the TAPSE is <2 standard deviation of age-related value	Single dimension, does not take into account of the circumferential or radial function of the RV. Alignment can be a problem	^320,321^
			Poor correlation with RV function and survival	
PAAT (ms)	Abnormal PAAT values with z-score <-2 SD were predictive of PH	Can easily be measured in all patients	HR-dependent	^256,322^
RV TDI E’ (cm/s)	<8 cm/s may indicate increased risk of mortality	Can easily be measured in all patients	HR-dependent	^323^
RV TDI MPI	Abnormal TDI MPI values indicates right ventricular dysfunction	Can easily be measured in patients	HR-dependent	^324^
S/D ratio	>1.4 indicates severity of PH and increased risk of mortality	Can easily be measured from TR velocity	Presence of defined TR velocity in systole and diastole	^272^
End-systolic RV/LV ratio	>1 may indicate increased risk of adverse events in pediatric PH	Easy to obtain clinically	Cannot be used in patients with left to right shunts	^325^
RV FAC (%)	Decreased FAC (<35%) correlates with decreased systolic function	Clinically easy to obtain	Does not take into account of the entire right ventricle	^324^
3D RV EF (%)	Decreased RV EF (<45%) indicates decreased RV function	Full volume datasets to evaluate for RV volumes and function; accurate measurements of RV function and can be prognostic in PH	Required breath-holding for adequate volumes; can be difficult even in single beat acquisitions when the pediatric probe is too big	^268,326^
RV strain (%)	RV free wall longitudinal strain decrease indicates decreased systolic RV function	RV free wall longitudinal strain maybe more sensitive in detecting ventricular function and is prognostic in PH	May not have adequate frame rate when the heart rate is too high	^273^
LV EF (%)	LV EF may be decreased in severe PH via RV-LV interaction	Quantification of LV function	Bi-plane Simpson’s may not be accurate as the LV is distorted in severe PH	^324^

TR, tricuspid regurgitant; RVSP, right ventricle systolic pressure; SPAP, systolic pulmonary arterial pressure; V, velocity; RA, right atrium; PH, pulmonary hypertension; mRAP, mean right atrial pressure; mPAP, mean pulmonary arterial pressure; RAP, right atrial pressure; DPAP, diastolic pulmonary arterial pressure; PAAT, pulmonary arterial acceleration time; SD, standard deviation; TDI, tissue Doppler imaging; MPI, myocardial performance index; FAC, fractional area change; RVEF, right ventricle ejection fraction; LVEF, left ventricular ejection fraction.

**Table 4.** Echocardiographic views to obtain functional parameters in pediatric PH.

	View	Functional parameters
Anatomy	Parasternal short axis	End-systolic RV/LV ratio^325^
Doppler	Parasternal short axis	Pulmonary acceleration time/ejection time
		Pulmonary regurgitation early and late diastolic velocity
	Apical four-chamber	Tricuspid regurgitation severity (none, mild, moderate, severe)
		Tricuspid regurgitation peak velocity
		Myocardial performance index (tricuspid tissue Doppler)^327^
		Systolic/diastolic (S/D) duration ratio from TR Doppler^272^
		Tissue Doppler systolic and diastolic velocities (tricuspid, septal, mitral)
Right atrium	Apical four-chamber	Right atrial area (RA)
Right ventricle	Apical four-chamber	Qualitative RV function (good, mildly/moderately/severely depressed)
		RV end-diastolic and end-systolic dimensions indexed for BSA (2D)
		Fractional area of change (FAC) %
		Tricuspid annular planar systolic excursion (TAPSE) with z-score (M-mode when available, 2D)
		RV strain (global and segmental)^43,326,328^
		3D RV volumes and function^268,326^
Left ventricle	Apical two- and four-chamber	LV ejection fraction (bi-plane Simpson or 5/6 area-length method)
		LV eccentricity index^317^
		LV strain (global)^329^
		3D LV volumes and function^324^
Other		Presence and severity of pericardial effusion (none, small, moderate, large)^231^

RV, right ventricle; LV, left ventricle; TR, tricuspid regurgitant jet; BSA, body surface area.

### 4.6 Cardiac magnetic resonance imaging in pediatric pulmonary hypertension

Cardiac MRI remains the gold standard imaging modality for assessment of bi-ventricular function and volumes in pediatric PH.^252^ MRI-derived functional and volumetric indices predict morbidity and mortality in pediatric PH and may provide additional information with respect to inter-ventricular interactions.^120,275^ In addition, phase-contrast MRI remains the state-of-the-art flow imaging technique enabling precise flow volume quantification and consequently provides valuable assessment of Q_p_/Q_s_ ratios in children with PH associated with congenital heart disease and intracardiac shunts. Furthermore, parallel or sequential phase-contrast MRI and pressure evaluation in the catheterization lab has been proposed as a novel and potentially more reliable method for the calculation of PVR in comparison to standard Fick principle or thermodilution.^276,277^ Recent studies suggest that pulsatile pulmonary vascular stiffness indices derived by phase-contrast MRI and ultrasound may have a strong prognostic potential to predict both hard and soft outcomes in pediatric PH.^278–280^ Lastly, MRA can aid with differential diagnosis by fine characterization of the pulmonary vasculature and exclusion of thrombi. Unlike echocardiography, cardiac MRI is currently not suitable for frequent serial assessment due to its clinical availability, longer post-processing time, and also due to the necessary anesthesia required for younger children and neonates.

In summary, children pose a different set of challenges to adult populations when it comes to imaging. Given the proven benefits of imaging adults with PH, it is appropriate that all patients including children are allowed to experience these benefits. Appropriate adaptations to imaging strategy and environment can achieve high-quality results in the vast majority of pediatric patients.

## Section 5 Future directions

### 5.1 Applications of computational modelling and artificial intelligence (AI) in pulmonary hypertension

#### Summary statements

1. Physiological modeling can be used to characterize the behavior of the cardiopulmonary system.
2. Computational models assessing PA flow have high diagnostic accuracy in suspected PH.
3. Machine learning approaches may assist image segmentation and improve diagnostic and prognostic assessments.
4. Further work to assess computational approaches versus current diagnostic approaches in PH is recommended.

Imaging modalities and markers derived based on images alone have been shown to have clinical potential. Their interpretation could be enriched by introducing additional knowledge from the application of mathematical models, which can bring insights into the hemodynamic system behavior in health and disease.

Based on mathematical and physical principles, models of the pulmonary circulation are currently being evaluated in the translational/clinical research. Electrical analogue (Windkessel or 0D-zero-dimensional) models supplied by patient-specific 2D phase-contrast MRI data have been proposed^281,282^ to characterize globally the pulmonary circulation in terms of vascular resistance and compliance in healthy and PH patients. They have also shown promising results for quantifying the changes of the electric parameters in patients with PAH, at baseline and follow-up.^283^ Wave transmission (one-dimensional [1D]) models are particularly powerful, as “waves carry information,” and the energy^282,284,285^ contained in the backward reflected wave can be used as an indicator between the right ventricle and its afterload mismatch. Using a 1D model of a straight elastic tube and temporal MRI flow and area waveforms, it has been shown that, on average, >40% of the total wave power was contained in the backward wave measured in patients with PH, whereas <20% was characteristic to the healthy volunteers group.^282^ Different PH heterogeneities can be mimicked by modifying the structural and elastic parameters of a 1D pulmonary tree structure. Several authors^285–287^ investigated the pressure and flow waveforms in healthy and several simulated PH conditions using numerical solutions of 1D models of the major vessel network. Notably, Qureshi et al.^285^ changed the configuration of the structured tree and altering, in turn, the compliance of the large and small vessels to predict the hemodynamic changes induced by PAH, CTEPH, and hypoxic lung disease. The authors showed an increase in the reflected waves under these simulated conditions, with the potential to differentiate between different PH phenotypes.

The 0D and 1D models have the main advantage that they are relatively simple to implement and do not require significant computing resources. However, 3D computational fluid dynamics (CFD) models are more complex, being able to provide patient-specific characterization of hemodynamics.^288,289^ Full 3D CFD simulations can resolve the physiological flow field in all three directions and time. Further post-processing of the CFD results can provide computed metrics (e.g. wall shear stress [WSS]), which give additional insights on disease progression. It has been argued^290^ that pathological flows in the PA alter cell behavior favoring vasoconstriction. 3D CFD^291,292^ studies of the pulmonary circulation showed that shear stress has an impact on endothelial health and dysfunction, and reduced WSS in the proximal PAs were shown to be characteristic to PH patients.

Machine learning of RV contours to derive tissue motion has shown to be of prognostic value in PAH and of greater significance than standard cardiac volumetric metrics.^293^ Such approaches are of great potential, minimizing user input/error and potentially providing a more complete prognostic assessment. The added value versus measurements adjusted for age, sex, and BSA,^118,294^ or standard CMR strain parameters in PAH is an area for further work. Machine learning approaches may significantly improve the automation and quantification of parameters from imaging. For example, deep learning approaches have already been utilized for automation of arteriovenous segmentation of the pulmonary circulation.^295^

The success of the computational models is closely related to the available data from the imaging modalities. Regardless of using the data only to supply the models’ boundary conditions, or integrate it within artificial intelligence algorithms,^296,297^ the computational models and imaging modalities play together an essential role in the process of non-invasive PH diagnostic, prognostic, and understanding of the disease mechanisms.

## Section 6 Conclusion and areas of research priority

In this statement, we have discussed recent advances in imaging techniques in PH and their clinical application. This is summarized in the PVRI Imaging Task Force diagnostic algorithm. Rapidly evolving technologies also provide opportunities to improve our understanding of PH and assess the impact of much-needed new therapies. This section identifies areas requiring further research in addition to highlighting a number of ongoing and planned studies.

### 6.1 Establishment of normative ranges and repeatability of imaging techniques

Despite the widespread use of imaging techniques in clinical practice, there are only limited data on the impact of age, sex, and ethnicity on commonly used metrics. The studies conducted to develop normative equations to allow for correction have been performed.^298–304^ However, given the significant impact of age, sex, and ethnicity on morphological characteristics, more research in this area is warranted to understand variation across populations.

There is increasing interest in using imaging endpoints in clinical trials to assess treatment response in the clinic setting. However, there are limited data on the repeatability of cardiac MRI measurements. The RESPIRE study (clinicaltrials.gov), which should report in the third quarter of 2019, will provide information on the sensitivity to change relative to measurement repeatability of cardiac MRI morphological and functional data. This will aid the design of studies considering MRI as a primary endpoint. Even in the absence of such data, studies such as REPAIR (clinicaltrials.gov) are now using imaging to assess the impact of pharmacological interventions. The results of these studies are awaited with interest.

### 6.2 Comparison of imaging modalities and approaches

There are pros and cons of different imaging modalities with respect to diagnostic performance, repeatability, availability, exposure to ionizing radiation, acceptance to patients, and cost. Often new imaging modalities and/or approaches are introduced into clinical practice with limited data. The cost of conducting large comparative studies and the rapid advances in the underlying technology that may occur during the conduct of a study may impact negatively on decisions to conduct such comparative studies. However, there is a pressing need to perform such technology appraisals. An important area is the diagnosis of CTEPH, where there have been significant advances in the imaging of the pulmonary vasculature. Additional data are required to critically evaluate these new techniques and challenge current guideline approaches. Importantly, a number of studies are planned, including the prospective, multicenter, comparative phase III diagnostic trial CHANGE-MRI, a European multicenter study comparing functional MRI and VQ-SPECT. This study aims to recruit 1000 patients (clinicaltrials.org). The INSPIRE study is a pilot non-inferiority study comparing iodine subtraction mapping with VQ-SPECT in patients with suspected CTEPH (clinicaltrials.gov). A prospective study comparing the cost and utility of follow-up approaches using echocardiography and cardiac MRI in patients with PAH is highly desirable.

An additional important area of research is the optimization of initial diagnostic testing and follow-up based on imaging availability, particularly where imaging and invasive testing are limited

### 6.3 Combining imaging with other modalities (genetics and MRI-augmented right heart catheterization)

With advances in genetics and imaging, there is an opportunity to better understand genotype–phenotype associations that have the potential to aid clinical decision-making. Heterozygous mutations in the gene-encoding bone morphogentic protein receptor type 2 (BMPR2) are the most common genetic cause of PAH, occurring in ∼15% of cases,^185^ and have been associated with worse RV function on cardiac MRI.^305^ Bi-allelic mutations in the eukaryotic translation initiation factor 2 alpha 5 kinase 4 gene (EIF2AK4) are described in PVOD and PCH,^306,307^ which are important to diagnose given their worse prognosis and poorer response to PAH therapies. In a large international cohort study,^185^ patients with PVOD were diagnosed based on radiological criteria; however, a number of patients who were carriers of EIF2AK4 were not identified by imaging alone. These patients were younger and had a lower gas transfer factor and greater interlobular septal thickening and mediastinal lymphadenopathy. This suggests that combining imaging with genetic testing in at-risk patients has the potential to improve diagnostics and prognostication. Integration of genetics, clinical data, and imaging is an exciting area for further research.

Diagnosis and accurate prognostication in patients with pulmonary vascular disease remains challenging. Hemodynamic data from RHC provides important information with direct pressure measurement. However, it does not provide a complete morphological or functional assessment of the right ventricle and pulmonary circulation. MRI-augmented catheterization involves invasive RHC performed inside the MRI system.^276,308,309^ It combines simultaneous invasive hemodynamic and MRI morphological and functional assessment in a single radiation-free procedure, providing detailed physiological insights. Further work to investigate the potential advantages in terms of clinical utility, improved understanding of disease, patient acceptability, and cost is recommended.

### 6.4 Improving our understanding of pulmonary vascular disease by assessing the distal vasculature and using novel imaging approaches

A limitation of current imaging modalities in PH is the insensitivity to visualize and interrogate the distal pulmonary arterial vasculature, the primary site of disease in patients with PAH. Assessing pulmonary perfusion directly using novel MRI or CT methods may provide a significant insight to the underlying small vessel vasculopathy. Temporal and spatial heterogeneity of the blood flow in patients at baseline and during PAH therapy is an area for further research.

Inhaled Xe^129^ hyperpolarized gas imaging is an emerging technique. Reports of hyperpolarized gases in CTEPH have suggested a potential role in follow-up of patients^310^ and abnormalities in IPAH have also been reported.^311^ A patient inhales Xe^129^ gas and ventilation images are acquired. Xe^129^ possesses relatively high solubility in tissues and blood. The transit of the gas from gas to dissolved phase can be probed using MRI spectroscopy. This allows the acquisition of functional parameters characterizing the gas exchange as well as gas uptake by imaging the dissolved phase. By studying these Xe^129^ diffusion properties, various microstructure parameters including alveolar-volume ratio, blood–gas barrier thickness, and surface-to-volume ratio can be determined.

### 6.5 Stress imaging of the cardiopulmonary system

Imaging in patients with suspected PH is usually undertaken at rest but there is increasing interest in evaluating changes in PAP and RV function on exercise and following other acute interventions. Exercise echocardiography is well described^312–315^ in the assessment of patients with PH, but currently its use is not widespread. There is increasing interest in using CMR and augmented MRI RHC to assess cardiopulmonary system on exercise to determine the differential response of RV morphology and function in health and disease. Further evaluation of the effect of acute interventions such as fluid and acute vasodilator challenges would also be of value.

### 6.6 Artificial intelligence and applications in pulmonary hypertension

With the rapid development in machine learning technologies, there is huge potential to improve imaging assessments. Image acquisition, analysis, and interpretation are areas that may benefit from integration of AI technologies. There are a number of recent publications in this area.^293,296,297^ Notable areas for further research include acceleration of MRI acquisition, improved segmentation of the cardiac chambers on CT and MRI and the pulmonary vasculature on CT, and diagnostic and prognostic classification and interpretation.

### 6.7 The impact of imaging on patients and users

Much of the focus of imaging research is based on the knowledge that can obtained by the images themselves. However, an important area of investigation is the impact of imaging: on patients and their families, physicians and healthcare professionals, researchers who interpret the images, and healthcare systems that fund imaging. The acceptability, emotional impact, and cost of clinical pathways to diagnose and serially assess patients requires further research, including the relative tolerability, risks, and benefits of different imaging approaches. A study evaluated the social and technological epistemology of clinical decision-making as mediated by imaging.^316^ This study illustrated that images can fulfil a mediating role by aiding acquisition of knowledge and facilitating communication in addition to illustrating the highly social aspects of interactions with patients and within multidisciplinary meetings. Involvement of patients and their families and users of imaging is required if imaging is going to fulfil its potential.

## Conflict of interest

The author(s) declare that there is no conflict of interest.

## Funding

This study was supported by the Wellcome Trust [R/148654-11-1].
